# Severe thoracic spinal fracture-dislocation without neurological symptoms and costal fractures: a case report and review of the literature

**DOI:** 10.1186/1752-1947-8-343

**Published:** 2014-10-14

**Authors:** Bing Jiang, Runcheng Zhu, Qingyan Cao, Hong Pan

**Affiliations:** 1 Department of Orthopedic Surgery, Anqing Hospital, Anhui Medical University, 352th Renmin Road, Anqing City, Anhui Province 246003, China

**Keywords:** Dislocation, Fracture, Thoracic vertebrae

## Abstract

**Introduction:**

Only a high-energy force can cause thoracic spinal fracture-dislocation injuries, and such injuries should always be suspected in patients with polytrauma. The injury is usually accompanied by neurological symptoms. There are only a few cases of severe thoracic spinal fracture-dislocation without neurological symptoms in the literature, and until now, no case of severe thoracic spinal fracture-dislocation without neurological symptoms and without costal fractures has been reported.

**Case presentation:**

A 30-year-old Han Chinese man had T6 to T7 vertebral fracture and anterolateral dislocation without neurological symptoms and costal fractures. The three-dimensional reconstruction by computed tomography and magnetic resonance imaging indicated the injuries in detail. A patient with thoracic spinal fracture-dislocation without neurological symptoms inclines to further dislocation of the spine and secondary neurological injury; therefore, laminectomy, reduction and internal fixations with rods and screws were done. The outcome was good. Severe spinal fracture-dislocation without neurological symptoms should be evaluated in detail, especially with three-dimensional reconstruction by computed tomography. Although treatment is individualized, reduction and internal fixation are advised for the patient if the condition is suitable for operation.

**Conclusions:**

Severe thoracic spinal fracture-dislocation without neurological symptoms and costal fractures is frighteningly rare; an operation should be done if the patient's condition permits.

## Introduction

The thoracic spine is stable because of kyphotic alignment, rib cage and costovertebral joints. The spinal canal is narrow; there is little free space between the cord and the osseous ring. The thoracic spinal cord also has a relatively sparse blood supply. Therefore, any compression or kyphosis in the thoracic spine always causes spinal cord injury which is usually severe in the upper thoracic spine.

There are only a few cases of severe thoracic spinal fracture-dislocation without neurological symptoms in the literature [1-14]. Only a powerful force can cause fracture-dislocation of the thoracic spine concomitant with life-threatening injuries [15]. Severe upper thoracic spinal fracture-dislocation is usually accompanied by complete neurological dysfunction and multiple costal fractures, but a few patients with thoracic spinal fracture-dislocation present non-neurological symptoms and patients with thoracic spinal fracture-dislocation without neurological symptoms and costal fractures are frighteningly rare. Here we report the case of a patient with T6 to T7 vertebral fracture and gross anterolateral dislocation without neurological symptoms and costal fractures, and discuss the clinical and radiological features, mechanism of the injury, and the treatment of the thoracic spinal fracture-dislocation.

## Case presentation

**Figure 1:**
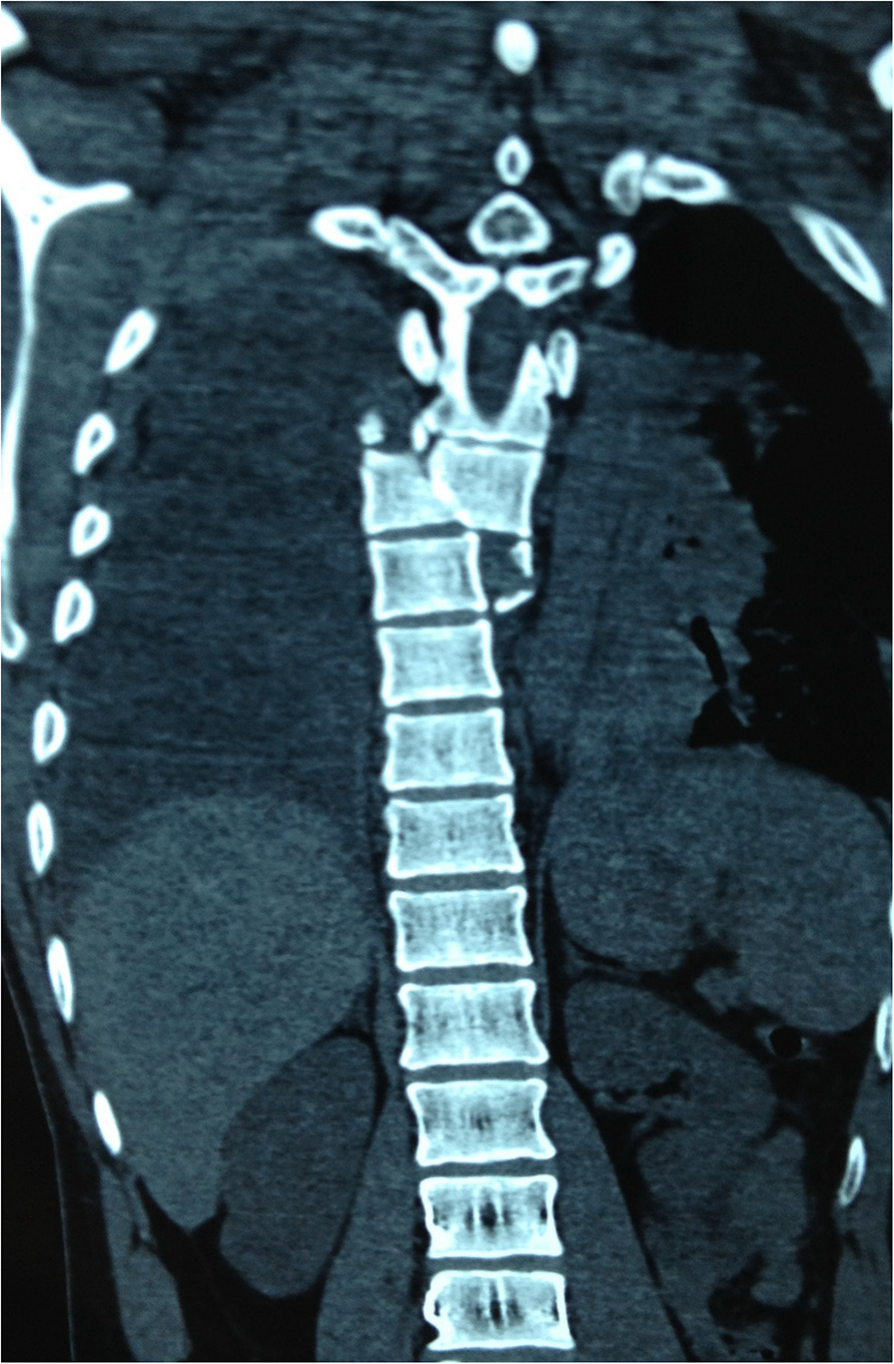
Three-dimensional reconstruction of computed tomography showed spinal fracture-dislocation of T6 to T7.

**Figure 2:**
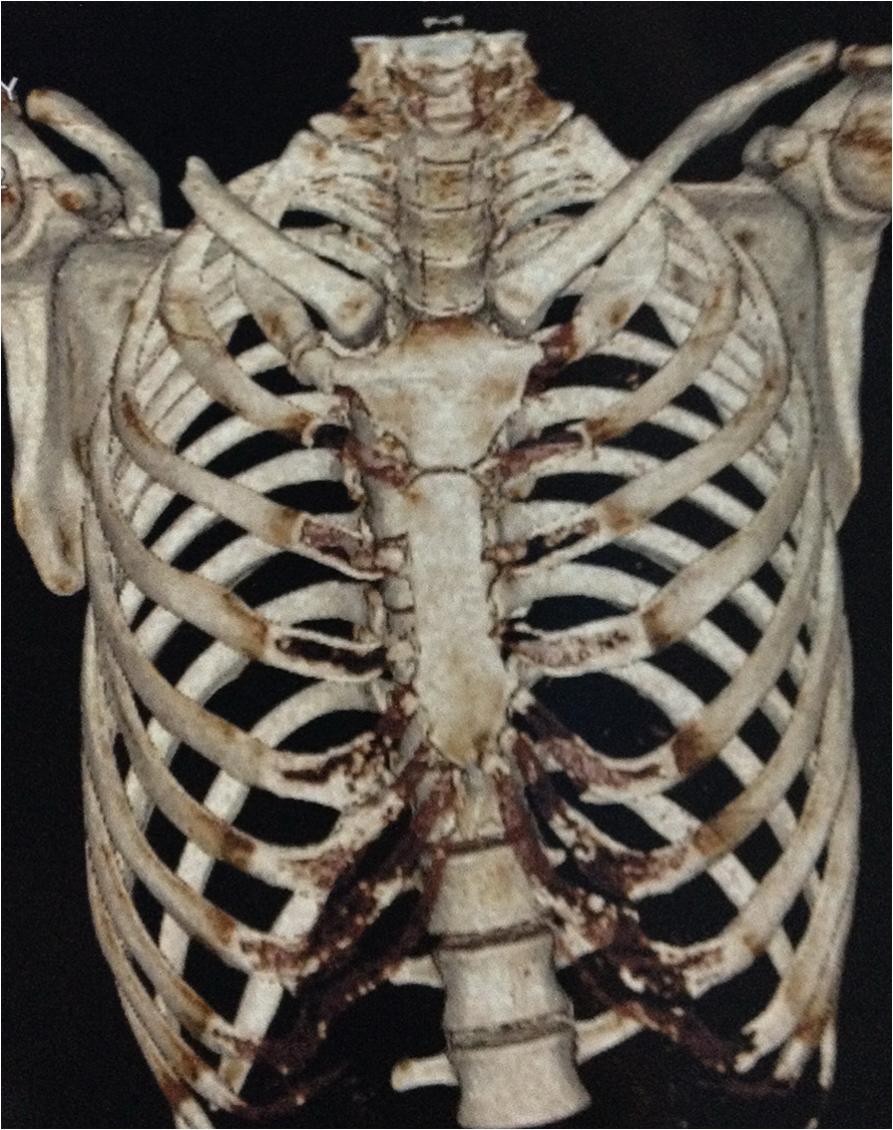
Three-dimensional reconstruction of computed tomography showed a fracture of right clavicle and non-fracture of ribs.

**Figure 3:**
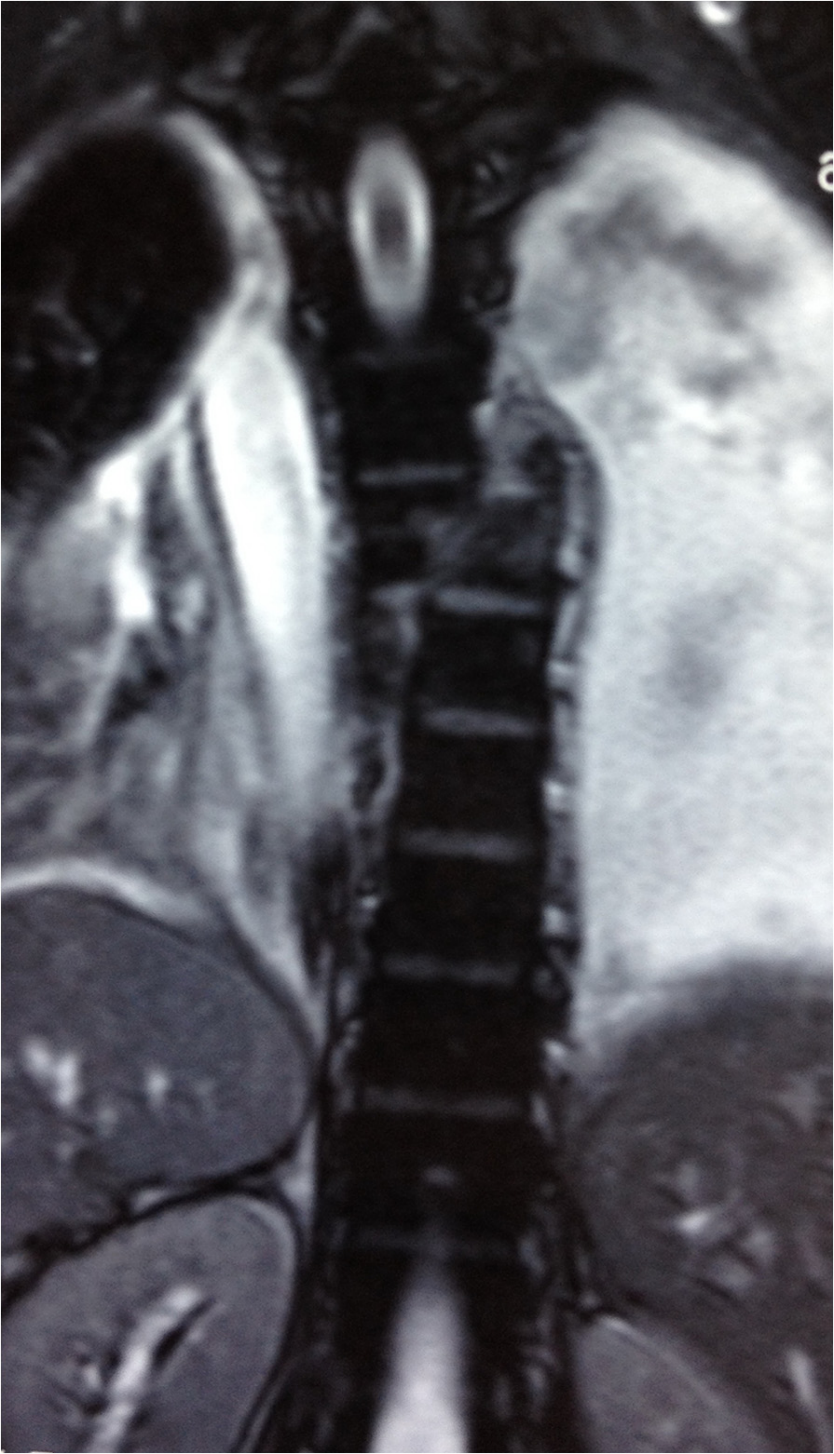
Magnetic resonance imaging showed spinal fracture-dislocation of T6 to T7.

**Figure 4:**
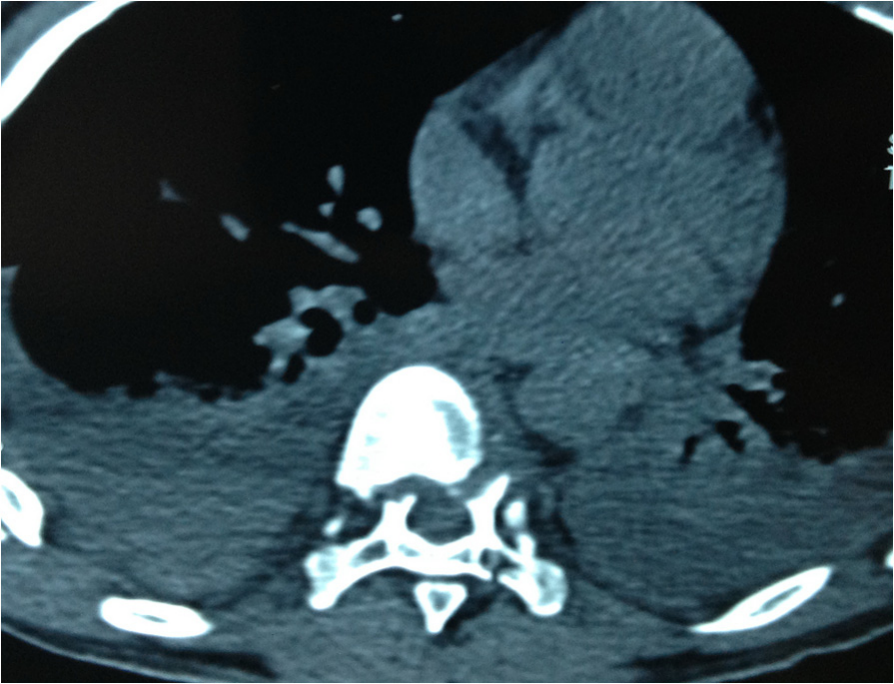
Horizontal computed tomography scans showed a large bilateral hemothorax; fractures of bilateral pedicles of vertebral arch.

**Figure 5:**
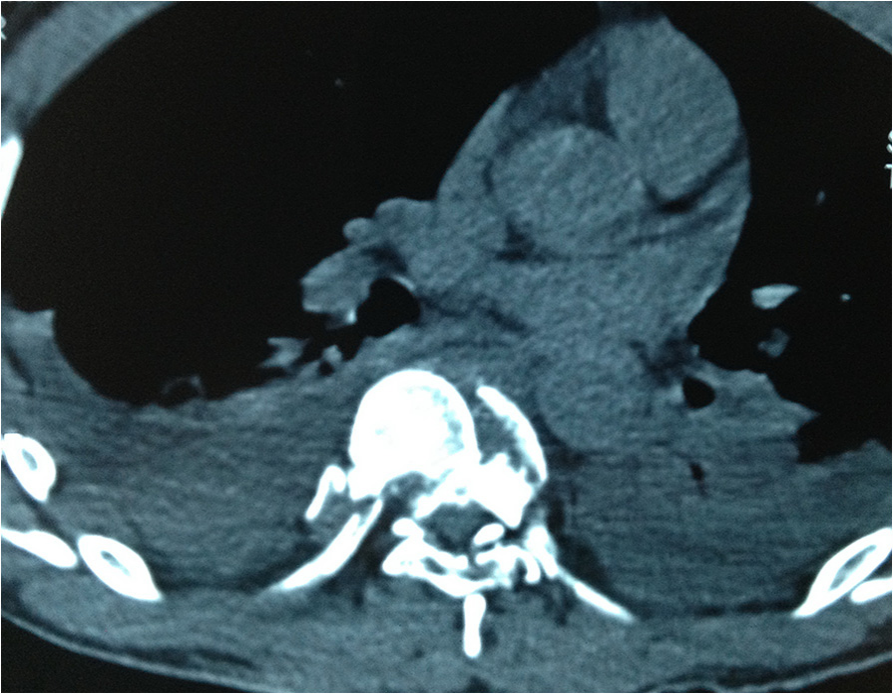
Horizontal computed tomography scans showed a large bilateral hemothorax; three vertebral levels in one cut.

**Figure 6:**
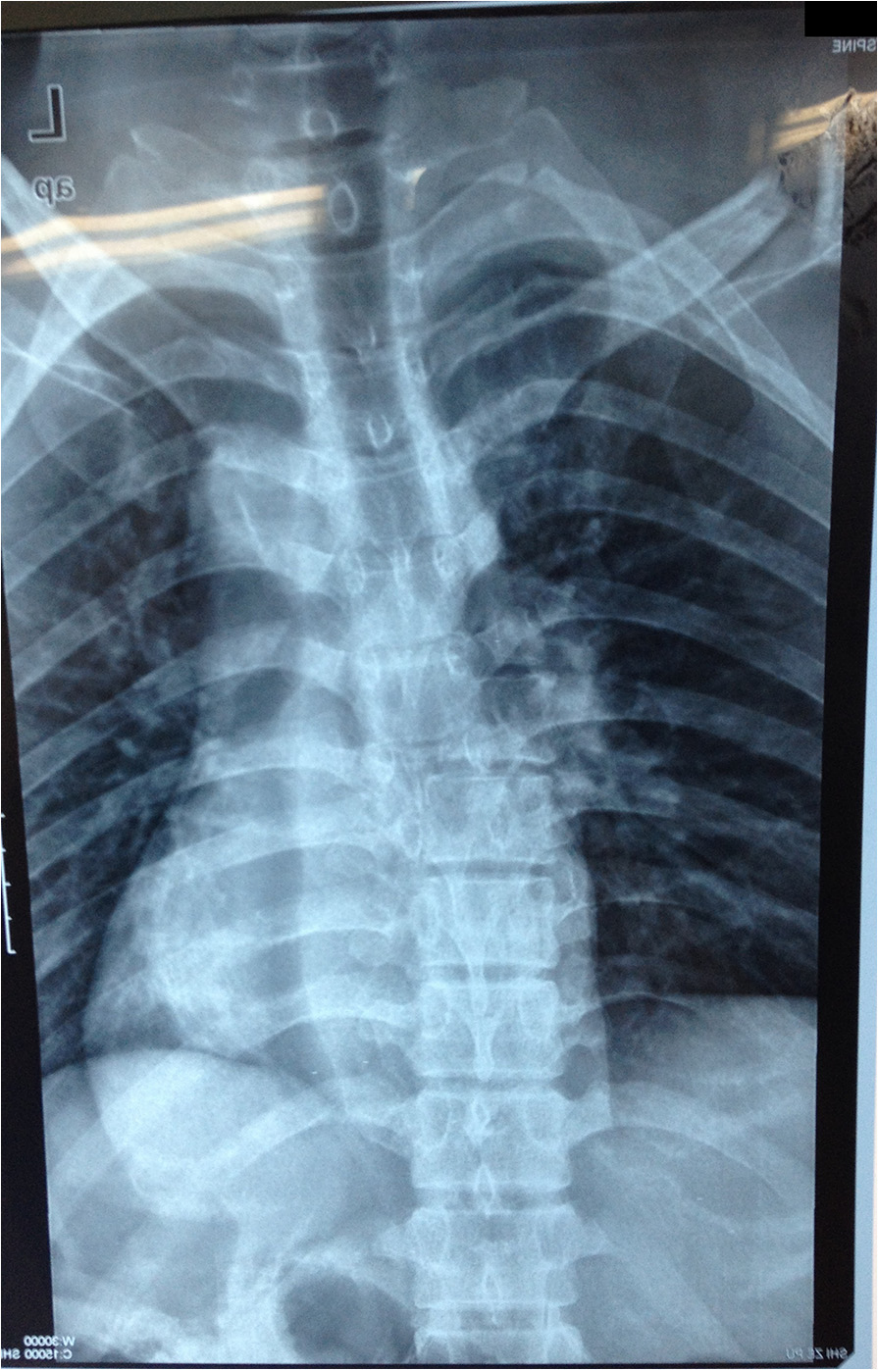
Anteroposterior thoracic spine plain X-ray showed spinal fracture-dislocation of T6 to T7.

**Figure 7:**
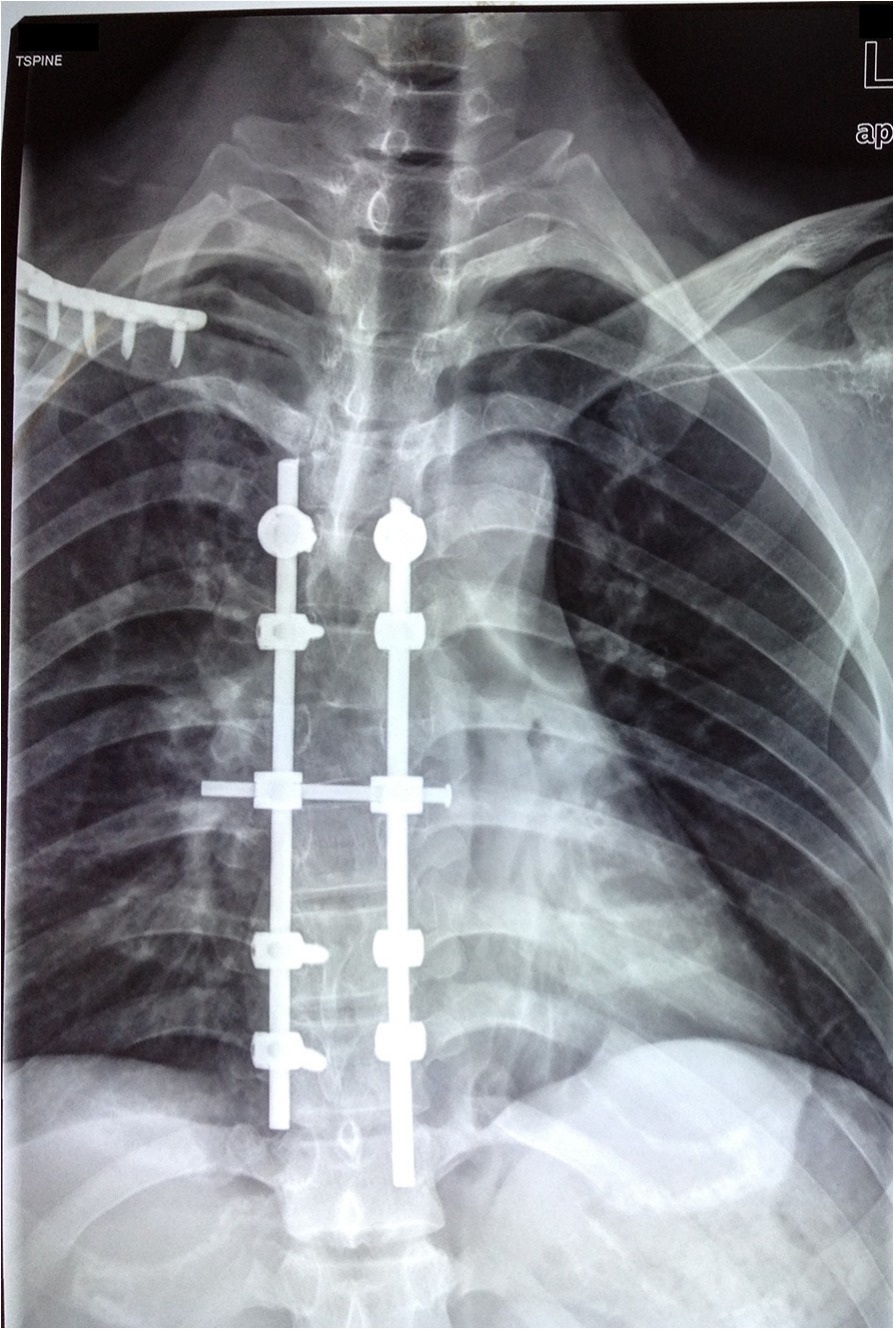
Anteroposterior thoracic spine plain X-ray showed realignment of the fractured-dislocated spine.

A 30-year-old Han Chinese man was riding his motorcycle at 80.5km/hour when he collided with an oncoming car. He complained of back pain and right shoulder pain and was admitted to a local hospital. He sustained double inferior lung contusion with a few hemothoraces, fractures of right clavicle and vertebrae T6 to T7 inclusive. After 6 hours, he was transferred to our hospital. On clinical examination, there was no neurological deficit. A three-dimensional reconstruction of computed tomography showed spinal fracture-dislocation of T6 to T7 (Figure 1), a fracture of right clavicle whereas fracture of ribs was not found (Figure 2), magnetic resonance imaging showed spinal fracture-dislocation of T6 to T7 (Figure 3), horizontal computed tomography scans showed a large bilateral hemothorax (Figures 4 and 5), fractures of bilateral pedicles of vertebral arch (Figure 4), three vertebral levels in one cut (Figure 5), and anteroposterior thoracic spine plain X-ray showed spinal fracture-dislocation of T6 to T7 (Figure 6) and realignment of his fractured-dislocated spine (Figure 7).

Despite the normal neurological systems below T7, the spine was considered unstable and an operation was planned. Chest drains were inserted bilaterally. A day later, the patient was put in the prone position under general anesthesia, the fascia was opened and his paravertebral muscles were dissected following a midline vertical incision of T4 to T11; the right pedicle of the T8 vertebra and the right lamina of the T6 vertebra were found to be fractured. Laminectomy of the T6 to T7 vertebra was done. The dura and spinal cord were found to be normal. Then the spinal canal was examined with a rubber catheter and found to be good. Transpedicular screws were put in the T5, T6, T9 and T10 vertebrae. Realignment of the thoracic spine was achieved with rods. Bony fusion of the transverse processes of the T5, T6, T7 and T8 vertebrae was done. The fracture of his right clavicle was also reduced and autologous bone graft was performed too. His nervous system was intact postoperatively. He was discharged on the 10th day after his operation. He was very well at the follow-up examination 3 months later.

## Discussion

Fracture-dislocation of the thoracic vertebrae without neurological symptoms is rare and no case involving severe thoracic spinal fracture-dislocation without neurological symptoms and costal fractures has been reported [1-14,16]. The thoracic spine is very stable because the articular facets of thoracic vertebrae are coronal and thoracic vertebrae connect to the ribs. Thoracic spinal fracture-dislocation occurs only when it is subject to strong violence, which usually accompanies spinal cord injury [17], lung contusion, pleural effusion, multiple costal fractures and other injuries. The injury mechanism in this case is complex; it is possible that powerful force was put on the right side of the patient’s body, causing fracture of his right clavicle, it continued to pass along his ribs to the thoracic vertebrae, resulting in fracture of thoracic vertebral and vertebral arch, then the fracture and dislocation of vertebral body is formed. As to the reason for his intact neurological function, spontaneous spinal decompression is considered to be the main mechanism [7]. To analyze the mechanism of the case: because of pedicle fractures to T7 and T8, the vertebral body (anterior column) of T7 and T8 shifted to the right, but the spinal cord and the posterior column of T7 and T8 did not shift, so spinal cord injury did not occur.

In Table 1, the key data of all 14 reported cases of thoracic fracture-dislocation with neural sparing have been compiled. Half thoracic fracture-dislocation with neural sparing occurred between T6 and T9 in half of the 14 reported cases, which is in accordance with the findings of Hanley and Eskay [18], this may be related to the fact that in the midthoracic area the spinous processes extend further inferiorly than in any other part of the thoracic spine [9].

**Table 1:** Date compiled from previously published cases of thoracic fracture-dislocation with neural sparing

**Case authors location**	**Level**	**Cause**	**Diagnosis**	**Pedicle fracture**	**Body fracture**	**Dislocation**	**Treatment**
1. Gertzbein anterolateral and Offierski [1],	T5/T6	Direct trauma	Immediate	Bilateral T6–T8	None	T5	Conservative
2. Vichard *et al.*[2], lateral	T8/T9	Car accident	Immediate	Right T8 left T9	None	T8	Conservative
3. Weber and Sutherland [3], lateral	T6/T7	Motorcycle accident	After 2 days	Bilateral T7–T9 right T10	Fractures through T7–T10	T6	Operative
4. Harryman [4], anterolateral	T6/T7	Car accident	Immediate	Bilateral T5–T8	None	T6	Operative
5. Sasson and Mozes [5], anterior	T9/T10	Car accident	Immediate	Bilateral T9–T10	None	T9	Operative
6. Uriarte *et al.*[6], anterolateral	T7/T8	Motorcycle accident	Immediate	Bilateral T7–T10	Horizontal shear fracture T7	T7	Conservative
7. Simpson *et al.*[7], anterolateral	T9/T10	Motorcycle accident	Immediate	Bilateral T9 right T7, left T8	None	T9	Operative
8. Krallis *et al.*[8], anterolateral	T7/T8	Motorcycle accident	Immediate	Bilateral T7	None	T7	Operative
9. Miyasaka *et al.*[9], T6 posterior and T8 anterolateral	T6/T8	Motorcycle accident	Immediate	Bilateral T9	Vertical fractures through T6–T8 and T10	T7	Conservative
10. de Lucas *et al.*[10], anterolateral	T8/T9	Car accident	Immediate	Right T8	Burst fracture T8, shear fracture T9	T8	Operative
11. Korovessis *et al.*[11], lateral	T5/T6	Motorcycle accident	After 6 weeks	Bilateral T5–T6	None	T5	Operative
12. Liljenqvist *et al.*[12], anterolateral	T9/T10	Car accident	Immediate	Left T9	Vertical fracture T9	T9	Operative
13. Potter *et al.*[13]?	T4/T5	Fall	Immediate	?	?	?	Operative
14. Anthes *et al.*[14], fractures T4 to T6	T4/T6	Motorcycle accident	Immediate	?	Comminuted anterior wedging T4	?	?

Table 1 indicates that the cause of most patients’ thoracic fracture-dislocation was a motor accident (12 out of 14). High-velocity impact with considerable violence may be the main pattern of injury leading to thoracic fracture-dislocation without neurological symptoms. In the case reported by Liljenqvist *et al.* the patient sustained severe spinal injury in a car accident while wearing a seatbelt [12].

Table 1 shows that fractures of the pedicle (12 out of 14 cases, and the other two are unknown) lead to separation of the posterior and middle column and avoidance of cord injury by maintaining the spinal canal. So fracture of the pedicle is a precondition for avoiding spinal cord injury.

To prevent our patient’s spinal cord from secondary injury and to realign his spine, surgery was carried out and a posterior spinal surgical approach was applied. To avoid iatrogenic injury of his spinal cord, laminectomy of the T6 to T7 vertebra was implemented firstly, then transpedicular screws were screwed in the T5, T6, T9 and T10 vertebrae and dislocation was corrected with rods. T7 and T8 were not fixed with screws because of fractures to vertebral pedicle. His clavicular fracture was also fixed and a bone graft was done. After the operation he recovered well. To avoid secondary injury to spinal cord, we should watch out for sharp broken bones in spinal canal and observe the tensity of spinal cord when decompression and reduction are performed.

As indicated in Table 1, an operative treatment was chosen in nine patients. Liljenqvist *et al.*[12] performed a posterior approach with two Roy-Camille plates and transpedicular screw fixation in T9, T10 and T11. De Lukas *et al.*[10] reported an anterior fusion without internal fixation and ambulated the patient in a cast after 2 months of bed rest. Weber and Sutherland [3] performed a combined anterior reduction and stabilization with AO plate and posterior internal fixation with two Luque rods. However, some considered the thoracic spinal fracture-dislocation to be stable [19] and conservative treatments such as halo traction were done and at follow-up it was reported that the patients were free from pain and were neurologically still intact.

Thoracic spinal fracture-dislocation without neurological symptoms and costal fractures is a rare and treatable clinical entity. It seems to be caused by a powerful force and may be accompanied by other injuries such as abdominal organ injury; we should examine the patient carefully and do some iconographical examinations in order to exclude other injuries and get a good grasp of the injury in detail [20]. Due to the relatively unstable thoracic spine of the fracture-dislocation, the spinal cord is extremely vulnerable and any movement may cause unintended neurological injury and further dislocation. So, the management principle for it is reduction and internal fixation if the condition is suitable for surgery, which allows early mobilization, enhances recovery, reduces the hospital stay, improves alignment of spine, prevents spinal cord from secondary injury, protects the spine from further dislocation, and results in fewer medical complications.

## Conclusions

Severe thoracic spinal fracture-dislocation without neurological symptoms and costal fractures is frighteningly rare; an operation should be done if the patient's condition permits.

## Consent

Written informed consent was obtained from the patient for publication of this case report and accompanying images. A copy of the written consent is available for review by the Editor-in-Chief of this journal.

## Competing interests

The authors declare that they have no competing interests.

## Authors’ contributions

BJ managed the patient, reviewed the literature and contributed to the preparation of the manuscript; RZ, HP and QC reviewed the manuscript and contributed to its final form. All authors read and approved the final manuscript.

## Authors’ information

Bing Jiang is an associate professor, Runcheng Zhu is a professor, Hong Pan MD is an associate professor and Qingyan Cao is an associate professor at Department of Orthopedic Surgery of Anhui Medical University affiliated to Anqing Hospital, China.
